# Elenbecestat and Compound 89 Potently Inhibit BACE1 but Not BACE2 When Subchronically Dosed in Non‐Human Primates

**DOI:** 10.1002/pmic.70082

**Published:** 2025-11-27

**Authors:** Sarah K. Tschirner, Andree Schmidt, Mana Ito, Kana Hyakkoku, Akimasa Yoshimura, Stephan A. Müller, Naotaka Horiguchi, Stefan F. Lichtenthaler

**Affiliations:** ^1^ German Center for Neurodegenerative Diseases (DZNE) Munich Germany; ^2^ Neuroproteomics, School of Medicine and Health TUM University Hospital Technical University of Munich Munich Germany; ^3^ Evotec München Neuried Germany; ^4^ Shionogi & Co., Laboratory for Drug Discovery and Disease Research Shionogi Pharmaceutical Research Center Toyonaka‐shi Osaka Japan; ^5^ Shionogi & Co., Vaccine R&D Laboratory Shionogi Pharmaceutical Research Center Toyonaka‐shi Osaka Japan; ^6^ Munich Cluster for System Neurology (SyNergy) Munich Germany

## Abstract

The β‐secretase BACE1 (β‐site amyloid precursor (APP) cleaving enzyme 1) is a major drug target for Alzheimer's disease (AD), as it catalyzes the first step in amyloid β (Aβ) generation, but has additional substrates and functions, in particular in the brain. Several advanced clinical trials with BACE1 inhibitors were stopped because of an adverse event, a mild cognitive worsening. The underlying mechanism is not yet known but may result from co‐inhibition of the BACE1‐homolog BACE2. While a cerebrospinal fluid (CSF) biomarker for measuring BACE2 activity is not yet established, VCAM‐1 has been suggested as such a biomarker, but has not yet been tested upon prolonged dosing in vivo. Using CSF pharmacoproteomics and a subchronic dosing paradigm in non‐human primates, we demonstrate that compound 89, a BACE inhibitor not yet tested in humans, and the clinically tested drug elenbecestat inhibit BACE1 in vivo, with little or no effect on BACE2, as seen with a reduction of substrates of BACE1, but not of the BACE2 substrate VCAM‐1. As a control, verubecestat, which inhibits both BACE2 and BACE1, reduced CSF abundance of BACE1 substrates as well as of VCAM‐1. This study demonstrates the suitability of VCAM‐1 as a pharmacodynamic biomarker for measuring BACE2 target engagement in CSF.

## 1 Main Text

Alzheimer's disease (AD) is one of the leading causes of dementia and death [1]. One intensively tested AD drug target is the β‐secretase BACE1 (β‐site amyloid precursor (APP) cleaving enzyme 1), one of two proteases involved in amyloid β (Aβ) peptide formation [2, 3]. Yet, several phase 3 BACE1 inhibitor clinical trials were terminated because of futility or the occurrence of a mild and reversible cognitive worsening [4], which needs to be prevented for potential future trials with BACE1‐targeted inhibitors. Because BACE1 cleaves numerous substrates besides APP and has fundamental functions in the brain, side effects may result from too strong inhibition of BACE1 and may thus be caused by functional alterations of BACE1 substrates [4]. Alternatively, the adverse event may stem from inhibition of BACE2, a close homolog of BACE1, which is co‐inhibited by all BACE1‐targeted inhibitors, but to a different extent [2, 4]. Monitoring BACE2 inhibition in vivo remains challenging. Given a function of BACE2 in pigmentation [5, 6], a fur greying assay, e.g., in mice, has been used in previous studies, but requires prolonged inhibitor treatment over weeks [5, 7, 8]. Recently, two BACE2 substrates were identified, which may allow for a faster and more accurate read‐out of changes in BACE2 activity in vivo. The BACE2‐cleaved ectodomain of vascular endothelial growth factor receptor 3 (VEGFR3) has been demonstrated as a plasma biomarker for BACE2 activity [7], while the cleaved ectodomain of vascular cell adhesion protein 1 (VCAM1, gene name; VCAM‐1, protein name) has been suggested as a biomarker in CSF [9]. Yet, VCAM‐1 has only been tested upon acute treatment after a maximum of two doses. It remains unclear whether VCAM‐1 is also suitable as a BACE2 activity biomarker upon prolonged inhibitor dosing.

To test the effect of subchronic dosing of BACE inhibitors on CSF biomarkers for BACE1 and BACE2 activity, we carried out two studies, in which non‐human primates (NHPs) were treated for 1 week with distinct BACE inhibitors. In the first study, compound 89 (also referred to as oxazine 89), a not yet clinically tested BACE inhibitor [10, 11], was enterally applied. Compound 89 has a 4‐fold selectivity towards BACE1 over BACE2, based on in vitro cleavage experiments [7]. In the second study, two BACE inhibitors (elenbecestat, verubecestat) were enterally applied, which were tested in phase 3 trials for AD [4]. In vitro, elenbecestat has a 3.5‐fold selectivity to BACE1 over BACE2 [4], while verubecestat has a 6‐fold selectivity for BACE2 over BACE1 [8].

**FIGURE 1:**
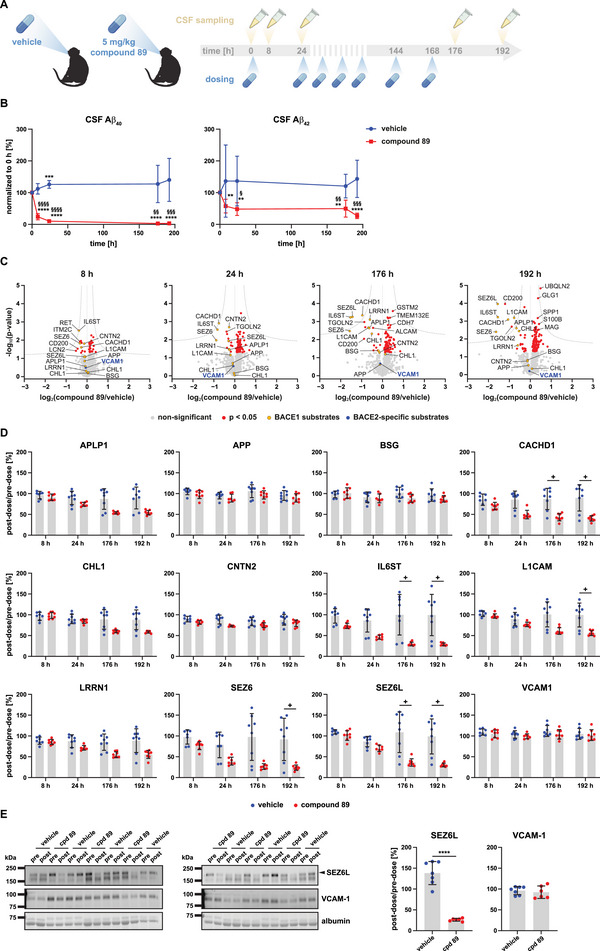
Subchronic treatment with compound 89 reduces Aβ_40_ and Aβ_42_ in NHP CSF without co‐inhibition of BACE2. (A) Cynomolgus monkeys were repeatedly treated with compound 89 or a vehicle control (*N* = 8 in each group) with a single dose per day over a period of 7 days. Pre‐dose CSF was sampled directly before treatment start (0 h). Post‐dose CSF was collected repeatedly at 8, 24, 176, and 192 h after treatment start. Created with BioRender.com. (B) CSF Aβ_40_ and Aβ_42_ levels in NHPs treated with compound 89 (*N* = 4–8) or a vehicle control (*N* = 8). For each animal, post‐dose values were normalized to the corresponding pre‐dose value. Pairwise comparisons of all post‐dose values versus pre‐dose (0 h) within each treatment group (*) and of matching post‐dose time points between the groups (§), as indicated in A. Unpaired, two‐sided Student's *t*‐test or one‐sample *t*‐test (when compared to 0 h) followed by correction for multiple testing by Benjamini‐Hochberg's FDR method. ^§/*^ *p* < 0.05, ^§§/**^ *p* < 0.01, ^§§§/***^ *p* < 0.001, ^§§§§/****^ *p* < 0.0001. (C) Proteomic analysis of CSF from NHPs treated with compound 89 (*N* = 7 in the 24 h group, otherwise *N* = 8) or vehicle (*N* = 7 in the 8 h group, otherwise *N* = 8). For each post‐dose time point indicated in (A), LFQ intensity values of each protein group and NHP were normalized to the corresponding pre‐dose value. In one volcano plot for each time point, post‐dose/pre‐dose ratios of the compound 89 group were compared to those of the time‐matched vehicle group by plotting log_2_ fold changes against –log_10_ *p* values, calculated by an unpaired, two‐sided Student's *t*‐test. Additionally, a permutation‐based FDR threshold was included, represented by dashed hyperbolic curves. Volcano plots include all protein groups detected in at least 6 replicates per group and in both the compound 89 and vehicle groups. BACE1 or shared BACE1/2 substrates are highlighted in yellow. VCAM‐1, which is selectively cleaved by BACE2, is depicted in blue. CHL1 was detected as two isoforms and, thus, is shown as two distinct protein groups (dots) in the plots. (D) Dot plots display post‐dose/pre‐dose ratios of LFQ intensity values for the BACE substrates from the dataset in (C). Plus signs indicate FDR‐significant differences between the treatment groups, as determined in (C). For CHL1, which was detected in two distinct protein groups, the protein group with most identified peptides was plotted. Data depict mean and SD. (E) Immunoblot detection of SEZ6L (the upper band marked with an arrow) and VCAM‐1 in pre‐dose (pre) and 192 h post‐dose (post) CSF from NHPs treated with compound 89 (cpd 89; *N* = 6) or vehicle (*N* = 7). Albumin was detected by total protein staining. Dot plots show immunoblot quantification results. Signals of each BACE substrate were normalized to the respective albumin signal, followed by calculation of the individual post‐dose/pre‐dose ratios. Data depict mean and SD. Unpaired, two‐sided Student's *t*‐test. ^****^ *p* < 0.0001. Only the significant difference is indicated.

In the first study, compound 89 or a vehicle control was applied subchronically over a period of 7 days with a single dose daily (Figure 1A). CSF was collected directly before treatment start and 8, 24, 176, and 192 h after the first dose, respectively (Figure 1A). To obtain a sufficient number of biological replicates (*N* = 8 per group), NHPs were treated repeatedly following a cross‐over dosing schedule (Figure S1A).

In the BACE1 inhibitor group, both CSF Aβ_40_ and Aβ_42_ levels progressively decreased over time with a final reduction of 97% for Aβ_40_ and 73% for Aβ_42_ after 192 h (Figure 1B), indicating excellent BACE1 target engagement. In the vehicle group, Aβ was not reduced. Next, CSF was proteomically analyzed via LC‐MS/MS and subsequent label‐free quantification (LFQ). For each NHP and treatment condition, LFQ intensity values of the different post‐dose time points were normalized to the corresponding pre‐dose values. Post‐dose/pre‐dose ratios of the compound 89 group were then compared to those of the time‐matched vehicle group, represented by volcano plots (Figure 1C). Known or putative BACE1 or shared BACE1/2 substrates [12, 13, 14, 15, 16, 17, 18] are depicted in yellow. VCAM‐1, which is cleaved by BACE2 but not BACE1 [9, 19], is shown in blue. Abundance changes for indicated, previously identified BACE substrates and substrate candidates are additionally visualized as dot plots showing the individual post‐dose/pre‐dose LFQ ratios (Figure 1D). As expected, the CSF abundance of BACE1 substrates gradually decreased over time. Effect sizes were strongest for “VWFA and cache domain‐containing protein 1” (CACHD1), interleukin‐6 receptor subunit β (IL6ST), seizure protein 6 homolog (SEZ6), and seizure 6‐like protein (SEZ6L), which, after 176 and 192 h, were all reduced by more than 50% relative to the vehicle control (Figure 1C,D). In contrast, compound 89 treatment did not affect VCAM‐1 abundance (Figure 1C,D), a recently identified CSF biomarker for BACE2 activity [9]. This indicates that compound 89 potently inhibits BACE1 but spares BACE2 in vivo. BACE1 preference of compound 89 was further confirmed by immunoblotting for SEZ6L and VCAM‐1, using the pre‐dose and 192 h post‐dose CSF samples (Figure 1E). In agreement with the proteomic data, immunoblot quantification showed a strong reduction for SEZ6L, whereas VCAM‐1 was unaltered between the post‐ and the pre‐dose samples (Figure 1E).

**FIGURE 2:**
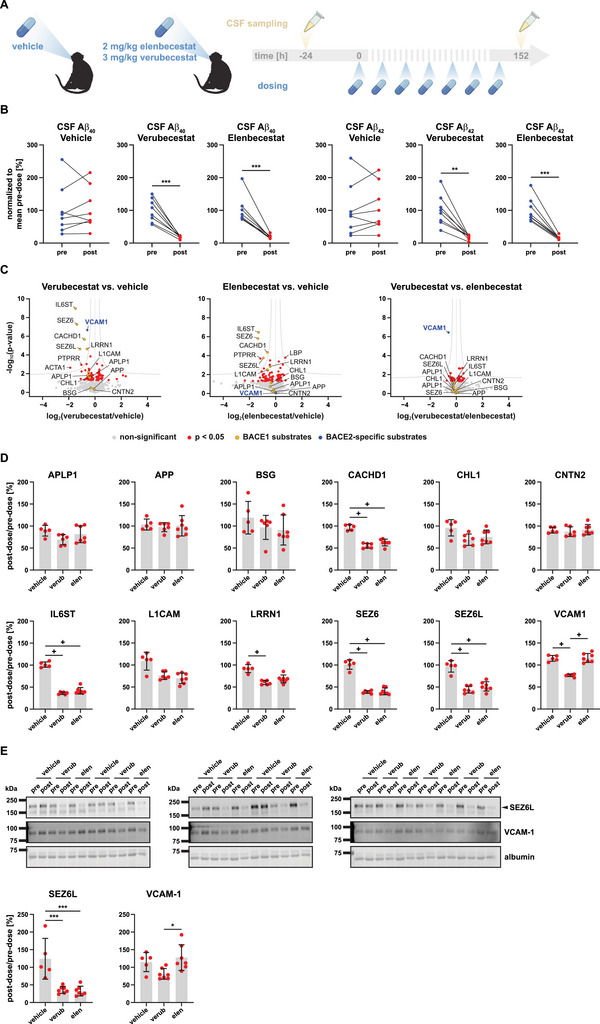
Subchronic administration of elenbecestat decreases Aβ_40_ and Aβ_42_ levels in NHP CSF without co‐inhibition of BACE2. (A) Cynomolgus monkeys were repeatedly treated with either verubecestat, elenbecestat, or a vehicle control (*N* = 8 in each group). Dosing was continued over a period of 7 days with an inter‐dose interval of 24 h. Pre‐dose CSF was taken 24 h before treatment start. Post‐dose CSF was sampled 152 h after treatment start. Created with BioRender.com. (B) CSF Aβ_40_ and Aβ_42_ levels in NHPs that were either treated with verubecestat, elenbecestat or a vehicle control (*N* = 8 in each group) before (pre) and after treatment (post). Paired, two‐sided Student's *t*‐test. ^**^ *p* < 0.01, ^***^ *p* < 0.001. Only significant differences are indicated. (C) Proteomic analysis of CSF from NHPs treated with verubecestat (*N* = 6), elenbecestat (*N* = 7), or vehicle (*N* = 5). Within each treatment group, post‐dose LFQ intensity values of each protein group and NHP were normalized to the corresponding pre‐dose value. Volcano plots show the comparisons of both inhibitor groups against vehicle treatment as well as against each other. For each comparison, log_2_ fold changes of the post‐dose/pre‐dose ratios of both groups were calculated and were plotted against –log_10_ *p* values obtained from an unpaired, two‐sided Student's *t*‐test. A permutation‐based FDR threshold was additionally included, shown as dashed hyperbolic curves. Volcano plots include all protein groups detected in at least 4 (vehicle), 5 (verubecestat), or 6 (elenbecestat) replicates, respectively. Substrates either selective for BACE1 or cleaved by both BACE1 and BACE2 are highlighted in yellow. The BACE2‐specific substrate VCAM‐1 is depicted in blue. APLP1 appears twice in the plots because it was detected in two distinct protein groups. (D) Dot plots display post‐dose/pre‐dose ratios of LFQ intensity values for the BACE substrates from the dataset in (C). Plus signs indicate FDR‐significant differences between the treatment groups, as determined in (C). For APLP1, which was detected in two distinct protein groups, the protein group with most identified peptides was plotted. Data depict mean and SD. (E) Immunoblot detection of SEZ6L (the upper band marked with an arrow) and VCAM‐1 in pre‐dose (pre) and post‐dose (post) CSF from NHPs treated with verubecestat (verub; *N* = 7), elenbecestat (elen; *N* = 7) or vehicle (*N* = 5). Albumin was detected by total protein staining. Dot plots show immunoblot quantification results. Signals of each BACE substrate were normalized to the respective albumin signal, followed by calculation of the individual post‐dose/pre‐dose ratios. Data depict mean and SD. One‐way ANOVA with Tukey's multiple comparisons test. Only significant differences are indicated. ^*^ *p* < 0.05, ^***^ *p* < 0.001.

In the second cross‐over subchronic dosing study, we treated NHPs with the BACE1‐preferring inhibitor elenbecestat in comparison to verubecestat, which blocks both BACE1 and BACE2, and a vehicle control (Figure 2A, Figure S1B). NHPs in each group were dosed once daily over a period of 7 days (Figure 2A). Pre‐dose CSF was taken 24 h before treatment start. Post‐dose CSF was collected 152 h after the first dose (Figure 2A). Similar to compound 89, CSF was subjected to determination of Aβ concentrations as well as LC‐MS/MS proteomic analysis.

**FIGURE 3:**
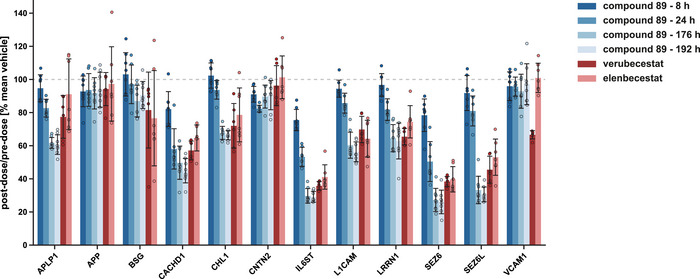
Effects of the BACE inhibitors compound 89, elenbecestat, and verubecestat on known or putative BACE substrates. The dot plot combines the CSF proteomics data of both the compound 89 and the elenbecestat/verubecestat study for the BACE substrates shown in Figure 1D and Figure 2D. Within each treatment group, post‐dose LFQ intensity values for the individual NHPs and BACE substrates were normalized to the corresponding pre‐dose value. For inter‐study comparability, post‐dose/pre‐dose ratios of the different BACE inhibitor groups were normalized to the mean post‐dose/pre‐dose ratio of the respective vehicle group. Data show mean and SD. Compound 89: *N* = 7 for 24 h, otherwise *N* = 8; elenbecestat: *N* = 7; verubecestat: *N* = 6.

Elenbecestat and verubecestat exhibited an equally potent inhibition of BACE1, as reflected by a reduction of ∼80% for both CSF Aβ_40_ and Aβ_42_, whereas vehicle treatment had no significant effect (Figure 2B, Figure S2). Aβ data are in line with decreased CSF abundance of the same set of BACE substrates as also seen for compound 89 (Figure 2C,D). Verubecestat, known to inhibit both BACE1 and BACE2, also led to a significant decrease of the BACE2‐specific substrate VCAM‐1, whereas this was not the case for elenbecestat (Figure 2C,D), which has a 3.5‐fold selectivity of blocking BACE1 over BACE2 in vitro [4]. Proteomic results were confirmed by immunoblotting for SEZ6L (as a BACE1 substrate) and VCAM‐1 (Figure 2E). Due to a higher variance within the vehicle group, VCAM‐1 immunoblot data for verubecestat compared to vehicle treatment did not reach statistical significance, but the effect sizes for all treatment groups were similar to the proteomic data. The comparable BACE1‐inhibitory and BACE2‐sparing profiles of compound 89 and elenbecestat are further visualized in an overall comparison of both studies in Figure 3.

To date, BACE1 preference of elenbecestat has been shown only in a cellular assay [4] but not yet in a quantitative approach in vivo. Our study demonstrates that both elenbecestat and the not yet clinically tested compound 89 potently inhibit BACE1 but do not appear to impact BACE2 activity when subchronically applied in NHPs. Moreover, our study reveals the suitability of the BACE2‐mediated cleavage product of VCAM‐1 as a CSF pharmacodynamic biomarker for monitoring BACE2 inhibition in vivo upon prolonged treatment. For potential future trials with BACE inhibitors, we propose to include additional biomarker measurements beyond the established ones, such as Aβ. For CSF analytics, we consider VCAM‐1 as a marker for BACE2 activity and SEZ6 and SEZ6L as markers for BACE1 activity. Measurement of these substrate/activity‐based markers for BACE1 and BACE2 may help to dose BACE inhibitors in a safe and individualized manner, allowing precision therapies in future trials for AD prevention or to prevent reoccurrence of amyloid pathology after Aβ immunotherapy [4, 20]. The biomarkers may also be instrumental to control dosing and side effects in trials for other diseases, including Down's syndrome, which is also accompanied by Aβ pathology in the brain [21, 22, 23], melanoma, glioblastoma, diabetes, and lung cancer, where BACE1 and BACE2 are also seen as potential drug targets [21, 22, 23, 24, 25, 26, 27]. Additionally, the biomarkers may allow the development of a new generation of BACE inhibitors that specifically target either BACE1 or BACE2.

## 2 Experimental Section

### 2.1 Animal Experiments

BACE inhibitors were applied in a cross‐over dosing schedule with four NHPs in total, as described in Figure S1. Inhibitors were dosed subchronically over a time course of 7 days, as shown in Figure 1A for compound 89 and in Figure 2A for verubecestat and elenbecestat. All NHPs (female, at an age of 8–15 years in the compound 89 study and 3–10 years in the elenbecestat/verubecestat study) had undergone experimental manipulation training beforehand. The animals were restrained in restraining cages (for less than 10 min), and drugs were administered enterally (intranasally and intragastrically) with a maximum administration volume of 5 mL/kg. For CSF collection in the elenbecestat/verubecestat study, NHPs were restrained in a restraining cage and were anesthetized with isoflurane. A syringe needle was then inserted into the cisterna magna, and a maximum volume of 0.5 mL CSF per time point was collected. In the compound 89 study, a volume of 0.25 mL CSF was withdrawn at each collection time point without anesthesia via an access port.

Animal experiments were approved by the Animal Care and Use Committee of the Shionogi Research Laboratories. Institutional Animal Care and Use Committee Approval No.: S15006C2‐0101 (compound 89 study), S20049C‐0001 (elenbecestat/verubecestat study).

### 2.2 CSF Aβ_40_ and Aβ_42_ Determination

Aβ_40_ and Aβ_42_ were quantified by using the Human β Amyloid (1‐40) ELISA Kit Wako (FUJIFILM Wako) and the Human β Amyloid (1‐42) ELISA Kit Wako, High Sensitive (FUJIFILM Wako), respectively, according to the manufacturer's instructions. For some samples, negative concentration values were obtained, which were excluded from further analysis.

### 2.3 CSF Proteomics

In the compound 89 study, 20 µL CSF was proteolytically digested according to the filter‐assisted sample preparation (FASP) protocol [28]. 10 kDa Vivacon 500 spin filters (Sartorius) were used. Tryptic peptides were desalted with C18 stop‐and‐go‐extraction (STAGE) tips [29] and were then dried by vacuum centrifugation. Peptides were reconstituted in 20 µL 0.1% formic acid and were then subjected to LC‐MS/MS analysis. CSF samples in the elenbecestat/verubecestat study were tested for blood contamination using Combur10‐Test strips [30]. Samples with a grade below 3+ were considered blood‐free. One post‐dose/pre‐dose pair of the elenbecestat group, one of the verubecestat group, and two pairs of the vehicle group were excluded from LC‐MS/MS analysis because of blood contamination of either the pre‐ or the post‐dose sample. From the remaining samples, a volume of 15 µL was subjected to single‐pot, solid‐phase‐enhanced sample preparation (SP3) [31] as previously described [9]. Reduced and alkylated proteins were bound to the beads in 70% acetonitrile while shaking for 30 min. After elution of the tryptic peptides, beads were sonicated for 30 s in 20 µL 0.1% formic acid. This second elution fraction was then pooled with the first one. Filtered peptides were dried and reconstituted as described above. Peptide concentration was determined by Qubit protein assay (Thermo Fisher Scientific). The overall proteomics workflow is depicted in Figure S3A.

In the compound 89 study, a sample volume of 5 µL was analyzed on a Q Exactive HF Hybrid Quadrupole‐Orbitrap mass spectrometer (Thermo Fisher Scientific), coupled to an EASY‐nLC 1200 nanoUHPLC system (Thermo Fisher Scientific) via a NanoFlex ion source with column oven (Sonation). Peptides were separated on an in‐house‐packed C18 column (30 cm × 75 µm ID, ReproSil‐Pur 120 C18‐AQ, 1.9 µm, Dr. Maisch GmbH) at a temperature of 50°C. A binary gradient was applied, composed of water (A) and acetonitrile containing 0.1% formic acid (B) at a flow rate of 250 nL/min. The gradient was as follows: 2% B at 0 min, 5% B at 2 min, 25% B at 92 min, 35% B at 112 min, 60% B at 121 min, 95% B at 123–138 min. Data were obtained by data‐independent acquisition (DIA) using the hyper reaction monitoring workflow [32], consisting of an MS1 spectrum at a resolution of 120,000 and 20 variable m/z windows at a resolution of 30,000. In the elenbecestat/verubecestat study, 300 ng of peptides were injected into a nanoElute HPLC system (Bruker) coupled online to a TimsTOF Pro mass spectrometer (Bruker) equipped with a CaptiveSpray ion source. Peptide separation was achieved as described above. The column length was 15 cm. The binary gradient at a flow rate of 300 nL/min (with A and B as described above) was composed as follows: 2% B at 0 min, 5% B at 2 min, 24% B at 62 min, 35% B at 72 min, 60% B at 75 min. DIA was applied in combination with parallel accumulation serial fragmentation (PASEF). One MS1 scan was followed by 2 rows of 40 sequential DIA windows at a width of 23 m/z and with an overlap of 1 m/z. The overall m/z range was 350–1200. The ramp time was fixed to 100 ms, and 4 windows were scanned per ramp, resulting in a total cycle time of 2.1 s.

Mass spectrometric data were processed in DIA‐NN (version 1.8) [33] and were further analyzed (Pearson correlation coefficients and subcellular location analyses as quality controls, volcano plots, dot plots for selected BACE substrates) as described previously [9] and in the figure legends. In DIA‐NN, a precursor m/z range of 300–1800 was applied. Quality control results are shown in Figure S3B (compound 89 study) and Figure S3C (elenbecestat/verubecestat study).

### 2.4 Immunoblotting

A volume of 18.5 µL CSF was subjected to SDS‐PAGE on 8% polyacrylamide gels and immunoblotting as described previously [9]. Immunoblots were developed by fluorescence imaging. Membranes were probed with the following primary antibodies: anti‐VCAM‐1 (AF809, R&D Systems) and anti‐SEZ6L (AF5598, R&D Systems). VCAM‐1 was detected with a fluorophore‐conjugated anti‐sheep secondary antibody (Alexa Fluor 680, A‐21102, Invitrogen). For SEZ6L detection, a fluorophore‐coupled anti‐goat secondary antibody (Alexa Fluor Plus 800, A32930, Invitrogen) was used. Albumin detection by total protein staining and quantification of SEZ6L and VCAM‐1 signals was performed as previously described [9].

## Author Contributions

S.K.T. analyzed the data, performed the immunoblot experiments, and wrote the first draft of the manuscript. A.S. and S.A.M. performed the mass spectrometric experiments. Shionogi (M.I., K.H., A.Y., and N.H.) conducted the animal work and determined and analyzed CSF Aβ concentrations. S.F.L. conceived the study together with Shionogi, analyzed the data, and wrote the manuscript. All authors revised the manuscript, data analysis, and interpretation of the data.

## Funding

This work was funded by the Deutsche Forschungsgemeinschaft (DFG, German Research Foundation) under Germany's Excellence Strategy within the framework of the Munich Cluster for Systems Neurology (EXC 2145 SyNergy—ID 390857198), with funding from the Federal Ministry of Research, Technology, and Space through CLINSPECT‐M (FKZ03LW0246) and with an Alzheimer's Association grant (SG‐23‐1029755 BACE).

## Conflicts of Interest

The authors declare no conflicts of interest, except M.I., K.H., A.Y., and N.H., who are employees of Shionogi.

## Supporting information




**Supporting Figure 1**: pmic70082 sup 0001 Figures.zip.

**Supporting Table 1**: pmic70082 sup 0002 Tables.zip.

## Data Availability

The mass spectrometry proteomics data have been deposited to the ProteomeXchange Consortium via the PRIDE [34] partner repository with the dataset identifiers PXD067461 (compound 89 study) and PXD067413 (elenbecestat/verubecestat study).
