# Supplementary material for: Elenbecestat and Compound 89 Potently Inhibit BACE1 but Not BACE2 When Subchronically Dosed in Non‐Human Primates
Source: Proteomics. 2025 Nov 27;26(1):100–7. doi: 10.1002/pmic.70082 (PMC12809003; doi:10.1002/pmic.70082)
Supplement: Supplementary file 1 — Supporting Figure 1: pmic70082 sup 0001 Figures.zip. [file PMIC-26--s001.zip › Supplementary Figures.pdf]

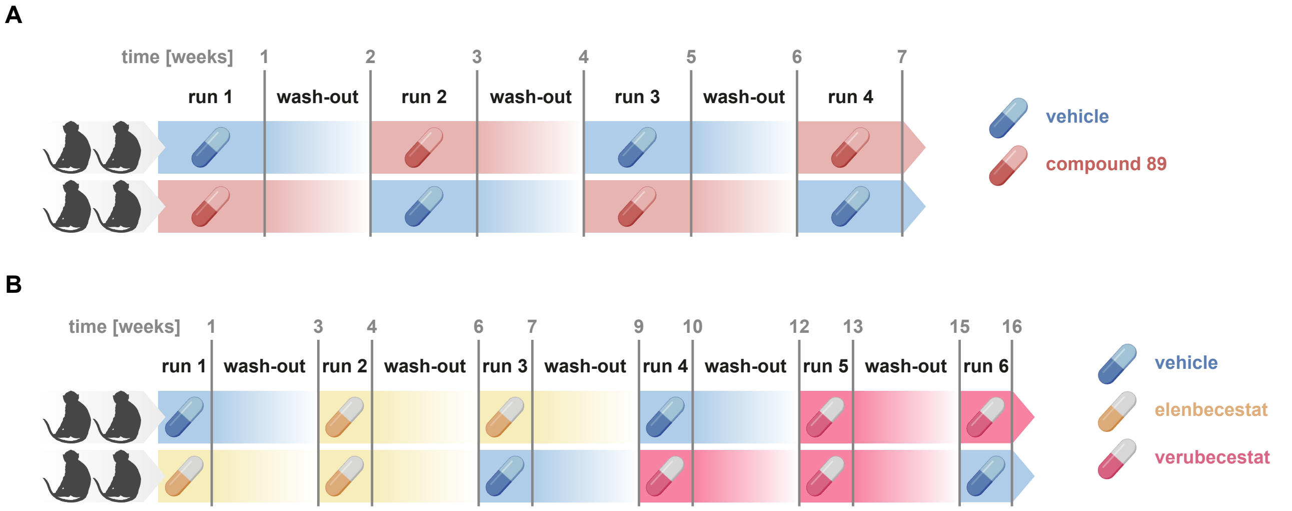

Supplementary Figure 1. Cross-over study design. A) Cynomolgus monkeys (N = 4 in total) were divided into two groups of N = 2. The animals in one group were treated with compound 89, while the NHPs in the other group received the vehicle control. Treatment was continued over a period of 7 days and was followed by a wash-out phase of 7 days. Afterward, groups were switched so that the former vehicle group next received compound 89 treatment and vice versa. In total, the NHPs underwent 4 runs of alternating treatment with a wash-out period between all runs. Drug dosing and CSF collection in each run was performed as shown in Fig. 1A, resulting in a final number of biological replicates of N = 8 for each treatment and sampling time point. B) NHPs (N = 4 in total) were divided into two groups of N = 2. All NHPs underwent 6 runs of drug treatment over a period of 7 days, separated by wash-out periods of 2 weeks. Drug treatment differed between the groups and was switched between runs. One group sequentially received the following treatment: vehicle, elenbecestat, elenbecestat, vehicle, verubecestat, verubecestat. In parallel, the second group was administered with: elenbecestat, elenbecestat, vehicle, verubecestat, verubecestat, vehicle. In total, each NHP received two treatment runs of elenbecestat, verubecestat and vehicle. Dosing and CSF collection was performed as described in Fig. 2A, resulting in a final number of biological replicates of N = 8 for each treatment and sampling time point. The figure was created with BioRender.com.

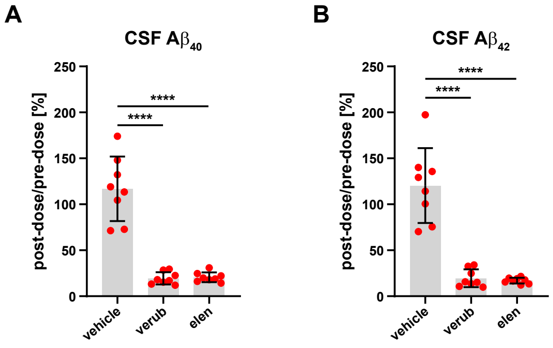

Supplementary Figure 2. CSF Aβ_{40} and Aβ_{42} levels are reduced in NHPs upon subchronic treatment with verubecestat or elenbecestat. Individual post-dose/pre-dose ratios were calculated for A) the CSF Aβ_{40} and B) Aβ_{42} data displayed in Fig. 2B. One-way ANOVA with Tukey's multiple comparisons test. Only significant differences are indicated. **** $p < 0.0001$. Data depict mean and SD.

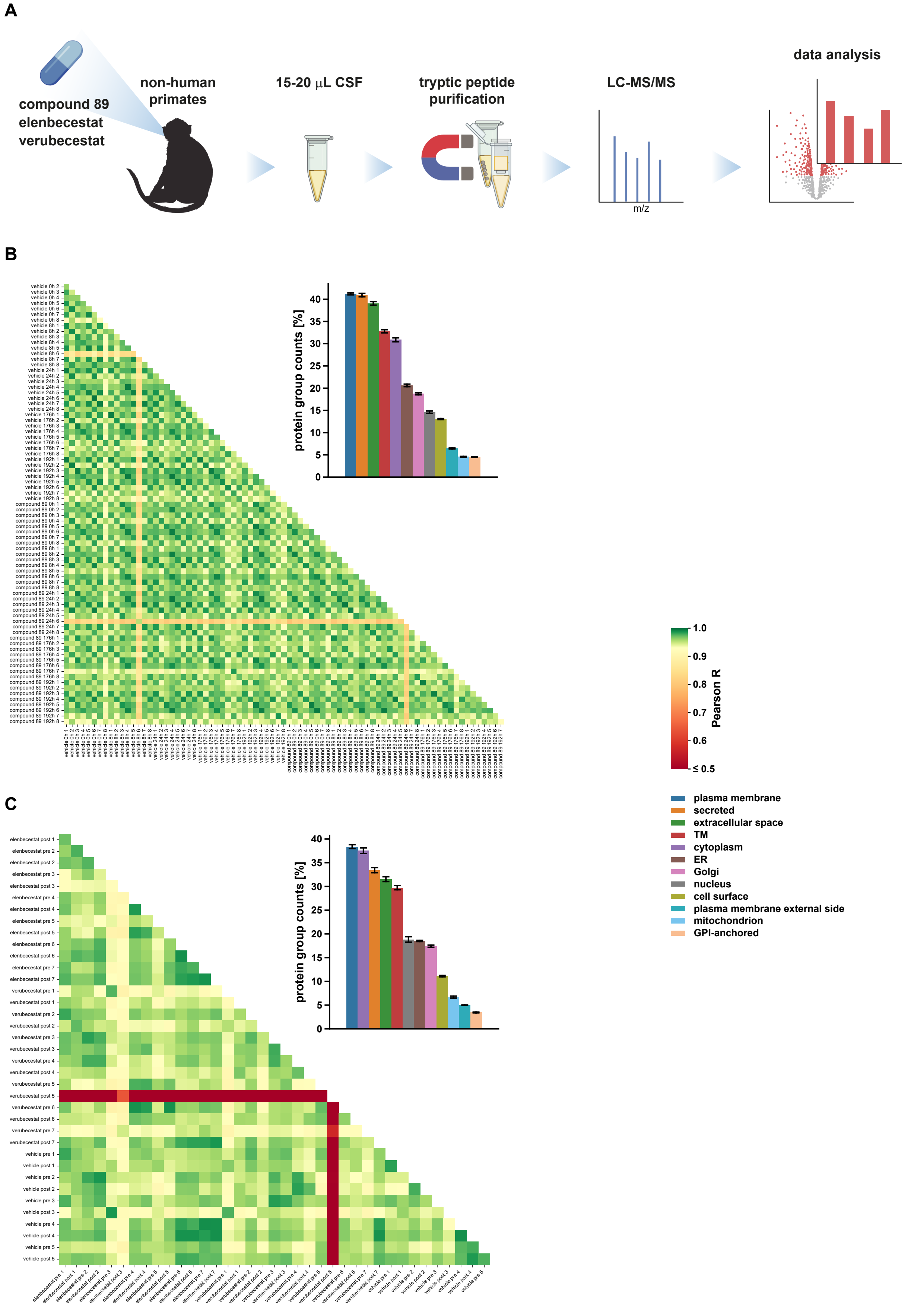

Supplementary Figure 3. NHP CSF proteomics workflow and quality control. A) Cynomolgus monkeys were treated with one of the BACE inhibitors compound 89, elenbecestat and verubecestat or were dosed with vehicle instead. Pre- and post-dose CSF was collected and digested with trypsin. Purified tryptic peptides were analyzed by LC-MS/MS. The figure was created with BioRender.com. B) Quality assessment for CSF proteomics data of the compound 89 study. Pearson correlation (R) matrix for log_2 LFQ intensity values of all protein groups that were detected with at least 2 peptides and in both samples of the respective pairwise comparison. A Pearson R of 0.93 and above for most comparisons indicates an overall good quality of the mass spectrometric data. Replicate 6 of the vehicle 8 h post-dose group and replicate 6 of the compound 89 24 h post-dose group appeared as outliers with Pearson correlation coefficients below 0.9 and were therefore excluded from downstream analyses shown in Fig. 1B and C.
Protein groups detected with at least 2 peptides and in a minimum of 75% of all samples were further analyzed for their subcellular location, depicted as bar plot with mean and SD. Subcellular location information was retrieved from UniProt for the human proteome (reviewed proteins only, database downloaded on May 5th 2025). Human proteome data were

chosen due to incompleteness of the cynomolgus monkey (*Macaca fascicularis*) database. Potential differences of subcellular locations between the species are considered negligible. NHP protein groups were matched to the human proteins via the gene names. The analysis was performed for the UniProt and Gene Ontology annotation terms listed in the diagram legend. In addition to subcellular location annotations, the classification of proteins as transmembrane (TM) or GPI-anchored proteins was considered. For each annotation term, the percentage of assigned protein groups in relation to the whole CSF proteome is shown. ER: endoplasmic reticulum. The majority of protein groups was either classified as plasma membrane proteins, secreted proteins or as located in the extracellular space. Mitochondrial proteins, on the other hand, only constituted one of the three least abundant annotation groups. These results indicate high CSF quality with only negligible amounts of cellular contamination.

C) Quality assessment for CSF proteomics data of the elenbecestat/verubecestat study as described in B. Pearson correlation coefficients of 0.93 and higher were obtained for the majority of inter-sample comparisons. An exception was replicate 5 of the verubecestat post-dose group which was therefore excluded from downstream analyses shown in Fig. 2B and C. Besides a relatively high fraction of proteins assigned to be cytoplasmic, the overall abundance of the different annotation terms suggests an overall dominance of cell surface, secreted and extracellular proteins. The high percentage of apparently cytoplasmic proteins most likely at least partially results from multiple annotations assigned to one and the same protein group, as e.g. in case of several possible isoforms. Therefore, potential cellular contaminations are more accurately reflected by the amount of mitochondrial proteins, which are among the three least abundant annotation groups. Altogether, these results are indicative for high CSF quality with only negligible amounts of contamination by intracellular proteins.
